# Results of the gErman migraine PatIent Survey on medical Care and prOPhylactic treatment Experience (EPISCOPE)

**DOI:** 10.1038/s41598-022-08716-w

**Published:** 2022-03-17

**Authors:** Marie Groth, Zaza Katsarava, Marc Ehrlich

**Affiliations:** 1 Novartis Pharma GmbH, Nuremberg, Germany; 2 Evangelical Hospital Unna, Unna, Germany; 3 Department of Neurology, University of Duisburg-Essen, Essen, Germany; 4 EVEX Medical Corporation, Tbilisi, Republic of Georgia; 5 IM Sechenov First Moscow State Medical University (Sechenov University), Moscow, Russian Federation

**Keywords:** Headache, Migraine

## Abstract

Migraine affects about 12% of the worldwide population causing substantial personal and societal burden. Yet, migraine remains underdiagnosed and untreated. EPISCOPE was a web-based survey among a German migraine patient cohort to characterize the medical care and prophylactic treatment status aiming to identify unmet needs. Potential migraine patients were identified via an ID Migraine screener. Their socioeconomic background, medical care experience, acute medication use, as well as use and experience of migraine prophylaxis was assessed by a questionnaire. Data of 29,011 participants was collected. 21,504 participants were identified as migraine patients. Patients with a higher number of monthly migraine days experienced better medical care. However, even among chronic migraine patients, 54% were not consulting a physician, 30% did not feel well-informed about medication overuse and 48% had never tried prophylactic migraine treatment. Among patients receiving prophylactic migraine treatment, up to 33% were not satisfied with their prophylaxis due to insufficient efficacy. Taken together, EPISCOPE describes the largest German migraine patient cohort so far. The survey provides detailed and valuable insight into the current medical care and prophylactic treatment situation in a highly developed European country and identifies reasons why the medical care of migraine patients is still insufficient.

## Introduction

Migraine is a common headache disorder that affects numerous important areas of life, including physical and psychological health, career and interpersonal dynamics^1^. According to the Global Burden of Disease Report 2016 (GBD 2016) it ranks as the world’s second leading cause of healthy life lost to disability (YLD). Therefore, the socioeconomic impact of migraine is enormous. Nevertheless, migraine is commonly underdiagnosed and undertreated not only in poorer countries but also in wealthy nations such as Europe and North America^2–4^. In Germany, only 8–11% of migraine patients used triptans and less than 3% of patients with ≥ 5 monthly migraine days (MMD) received preventative treatment^2,5^. We sought to verify this and to identify in more detail the reasons for underdiagnosis and undertreatment of migraine patients in Germany by analysing data from the EPISCOPE study.

EPISCOPE was an online survey that gathered data from a large cohort of self-identified migraine patients in Germany. As the aim of this study was to analyse the medical care of migraine patients, individuals who did not consider themselves to have migraine were excluded from the analysis. The survey included diagnostic inquiry regarding migraine and further exploration regarding the patient journey from disease onset to diagnosis and utilisation of medical services, medication and non-pharmacological therapies for migraine. Here, we present data on the current status of diagnosis and treatment of migraine patients in Germany, including consultations and migraine-specific acute and preventative medications, as well as the reasons for underdiagnosis and undertreatment of migraine in Germany.

## Results

### Demographic data

**Figure 1:**
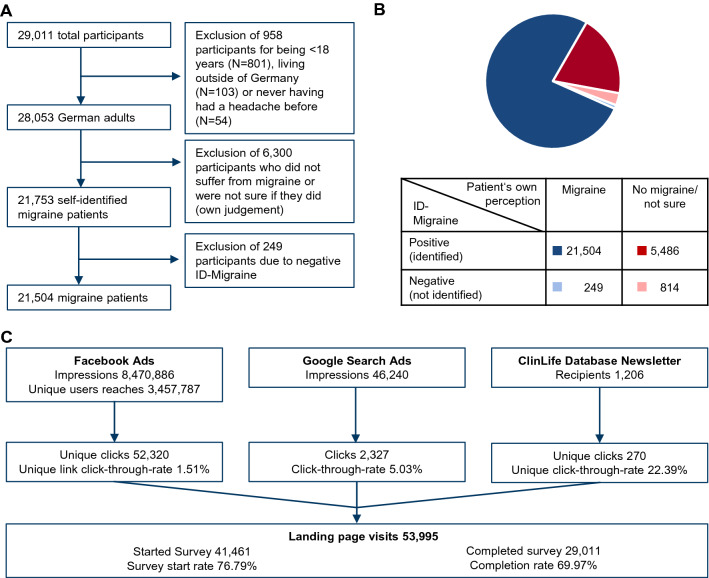
Description of survey participants. (**a**) Flowchart specifying the inclusion criteria of the survey. Taken together, 21,504 participants fit the criteria and will hereafter be referenced as “migraine patients”. (**b**) Graph showing disease awareness of survey participants. All German participants (N = 28,053) were categorised into aware of their migraine (N = 21,753, blues) vs. not aware/not sure (N = 6,300, reds) based on their own judgement. Assumptions were then confirmed via the ID Migraine screener. The screening was positive, and the patient was identified as a migraine patient if at least two out of three questions were affirmed (Supplementary Table S1). (**c**) Overview of the total view/click-through sample “n” and rate, participation “n” and rate, and completion “n” and rate for the different adverting channels.

From 53,995 landing page visits, 41,461 individuals started the survey, and 29,011 completed it (completion rate of 69.97%; Fig. 1C). Of 29,011 participants, 958 were excluded because they were younger than 18 years (N = 801), lived outside of Germany (N = 103) or had never had a prior headache (N = 54) (Fig. 1A). The remaining 28,053 participants underwent an ID Migraine screener, which consisted of three questions to validate whether the participant is indeed suffering from migraine (Supplementary Table S1). If two out of three questions were affirmed, a participant was identified as a migraine patient. According to their own judgement, 78% (N = 21,753) of all German participants considered themselves as migraine patients while 22% (N = 6300) stated they did not suffer from migraine or were unsure if they did (Fig. 1B). As we wanted to analyse the medical care of migraine patients, we excluded all participants from answering further questions who did not consider themselves as migraine patients. Amongst all participants who stated that they suffered from migraine, the subsequent well established ID Migraine screener identified 99% (N = 21,504) as potential migraine patients, resulting in the exclusion of an additional 249 participants from the study. The ID Migraine screener additionally revealed that of the 6,300 participants who stated that they did not suffer from migraine or were unsure if they did, the vast majority (87%, N = 5486) also suffered from migraine. If not specifically indicated, only patients living in Germany, initially stated that they were suffering from migraine and had a positive ID Migraine screener result (N = 21,504) were considered for the following analyses.

Most migraine patients, 85.5% (N = 18,390), were female, while 14.3% (N = 3077) male and 0.2% (N = 37) were diverse. The mean age of migraine patients were 29.2 ± 10.01 years and the majority was employed (71.4%, N = 15,345), while 1.7% (N = 356) had limited employment due to migraine, 0.8% (N = 161) were not able to work due to migraine, and 0.4% retired early due to migraine (N = 89) (Table 1).

**Table 1:** Demographic description of survey participants.

Characteristic	N = 21,504
**Age, years**	29.2 ± 10.01
**Gender**	
Female	85.5% (18,390)
Male	14.3% (3077)
Diverse	0.2% (37)
**Employment status**	
Employed	71.4% (15,345)
Student	12% (2585)
In school	6.4% (1375)
Retired	1.1% (232)
Limited employment due to migraine	1.7% (356)
Not working due to migraine	0.8% (161)
Early retirement due to migraine	0.4% (89)
Not working due to other reasons	6.3% (1361)
**Time since disease onset, years**	13.4 ± 9.9
**Monthly migraine days (MMD), days**	5.7 ± 5.4
< 4	46.1% (9920)
4–7	29.3% (6297)
8–14	15.4% (3313)
≥ 15	9.2% (1974)

Data are shown as mean ± SD or % (n).

### Clinical details

The mean time since migraine onset was 13.4 ± 9.9 years, and the mean monthly days (MMDs) in the three months before the survey was 5.7 ± 5.4 (Table 1). 46.1% (N = 9920) of patients reported < 4 MMD, 29.3% (N = 6297) 4–7 MMD, 15.4% (N = 3313) 8–14 days and 9.2% (N = 1974) reported ≥ 15 MMD.

### Medical care

**Figure 2:**
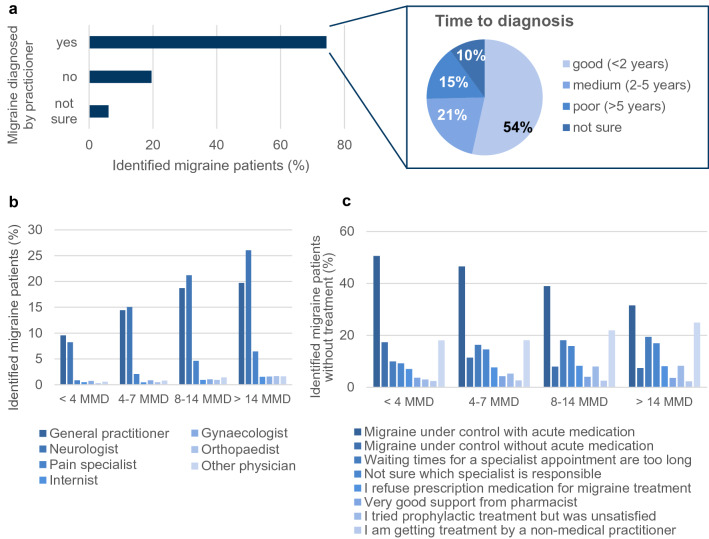
Medical care of migraine patients. (**a**) Left panel shows the proportion of identified migraine patients in the study population whose migraine was also diagnosed by a physician. Right panel shows the time to diagnosis. (**b**) Graph shows the types of consulted physicians depending on the patient’s MMD. Migraine patients were asked to indicate all doctors, whom they are currently seeing and in total, 5629 patients were seeing at least one doctor. (**c**) Graph indicates the patients’ reasons against seeking medical advice**.** Identified migraine patients who were not consulting a practitioner for their migraine at the time of the survey (N = 15,875) were asked for their reasons (indication of multiple reasons was allowed). Shown are the percentages of patients depending on their number of MMD.

Of all identified migraine patients in this survey, 74% (N = 15,994) were diagnosed by a physician (Fig. 2A). 54% (N = 8558) received the diagnosis within two years after onset of their migraine, 21% (N = 3379) of patients were diagnosed 2–5 years after migraine onset, and 15% (N = 2455) of patients received the diagnosis more than 5 years after symptom onset. Most diagnoses were made by neurologists (52%, N = 8256), followed by general practitioners (39%, N = 6198) and pain specialists (3%, N = 513) (Supplementary Figure S1A). The proportion of patients diagnosed within 2 years was highest among general practitioners, followed by internists and gynaecologists; neurologists, pain specialists and orthopaedists saw a higher proportion of patients diagnosed after more than 5 years (Supplementary Figure S1B).

Although 74% of identified migraine patients were diagnosed by a physician, only 26% (N = 5629) were receiving any kind of treatment at the time of the survey. Even among patients with ≥ 15 MMD only 43% (N = 842) visited a doctor (Supplementary Figure S1C). Migraine patients thereby mostly sought help from general practitioners and neurologists, and the percentage of patients seeing neurologists compared to general practitioners increased with the amount of MMD (Fig. 2B).

As main reasons against seeking medical advice, most patients stated that they had their migraine under control with or without usage of acute medication (Fig. 2C). While 51% (N = 4137) of patients with < 4 MMD managed their migraine with acute medication, only 32% (N = 357) of patients with ≥ 15 MMD were able to do so. Similarly, while 17% (N = 1420) of patients with < 4 MMD had their migraine under control even without acute medication, this applied to only 7% (N = 84) of patients with ≥ 15 MMD. Furthermore, 14% (N = 2145) of patients across all MMD groups considered the waiting time for a specialist’s appointment too long and 12% (N = 1934), especially younger patients, were not sure which specialist to consult (Supplementary Figure S1D, right panel).

### Acute medication

**Figure 3:**
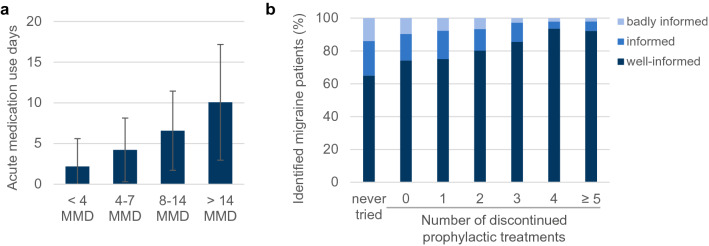
Acute medication use. (**a**) Graph depicts average number of days with acute medication days depending on MMD (mean +/− SD). (**b**) Graph shows the percentage of identified migraine patients that feel well-informed (dark blue), informed (blue), and badly informed (light blue) about medication-overuse depending on the number of discontinued prophylactic treatments. Total patients per group: never tried prophylactic treatment: N = 14,614; 0 discontinued: N = 3460; 1 discontinued: N = 2203; 2 discontinued: N = 657; 3 discontinued: N = 284; 4 discontinued: N = 94; ≥ 5 discontinued: N = 192.

More than 90% of all identified migraine patients used acute medication at least once per month with a similar number of acute medication days across gender and age (Supplementary Figure S2). The mean days of acute medication use per patient increased with rising number of MMD (Fig. 3A). When patients were asked about their knowledge of medication overuse, 69% (N = 14,758) stated “I know when medication overuse can occur and its risks”, while 19% (N = 4137) indicated “I have heard about medication overuse but I don’t know much about it” and 12% (N = 2609) declared “I know (almost) nothing about medication overuse” (Fig. 3B). Patients who had already tried multiple preventative treatments felt better informed than those who had never tried any prophylaxis (Fig. 3B). Similarly, patients with more MMDs had better knowledge about medication overuse than patients with fewer MMDs (Supplementary Figure S2D).

### Prophylaxis

**Figure 4:**
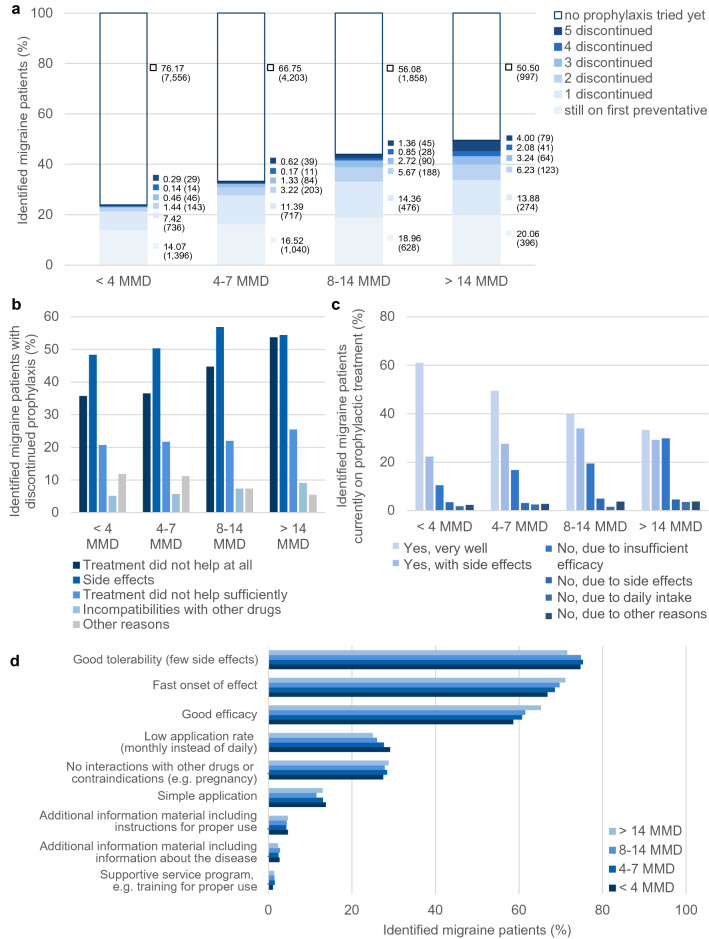
Prophylactic treatments. (**a**) Graph shows the percentages of identified migraine patients that have tried 0–5 different prophylactic treatments depending on their MMD; respective percentages are depicted in the graph (absolute number of patients in brackets). (**b**) Shown are reasons for discontinuation of preventive treatments depending on the patients’ number of MMD. All patients who had previously discontinued at least one preventive treatment (N = 3430) were asked for their reasons (multiple reasons per patient were allowed). (**c**) Graph shows satisfaction of identified migraine patients with their current prophylaxis depending on the number of their MMD. (**d**) Depicted are the patients’ demands on a new prophylactic migraine treatment depending on their number of MMD.

The number of migraine patients who had previously tried at least one preventative treatment increased with the number of MMD (filled blue stacks, Fig. 4A). However, even among patients with ≥ 15 MMD, half (51%, N = 997) had never tried prophylactic migraine treatment. Patients with more MMDs have cycled through a higher number of different prophylaxes than patients with fewer MMD. The main reasons for discontinuation were thereby the occurrence of side effects, ranging from 48% (N = 468) among patients with less than four MMD to 54% (N = 316) in the group with greater than or equal to 15 MMD, and lack of efficacy, which ranged from 36% (N = 346, 4 < MMD) to 54% (N = 312, ≥ 15 MMD) (Fig. 4B). In addition, among patients who were still on prophylactic treatment at the time of the survey, between 10% (patients with < 4 MMD) and 30% (patients with ≥ 15 MMD) were not satisfied with their current prophylaxis due to insufficient efficacy (Fig. 4C).

When asked about their three most important demands on a new prophylactic treatment, most patients named good tolerability (75%, N = 16,043), fast onset of effect (68%, N = 14,657) and good efficacy (60%, N = 12,963) (Fig. 4D).

### Drug-free prophylactic treatments

**Figure 5:**
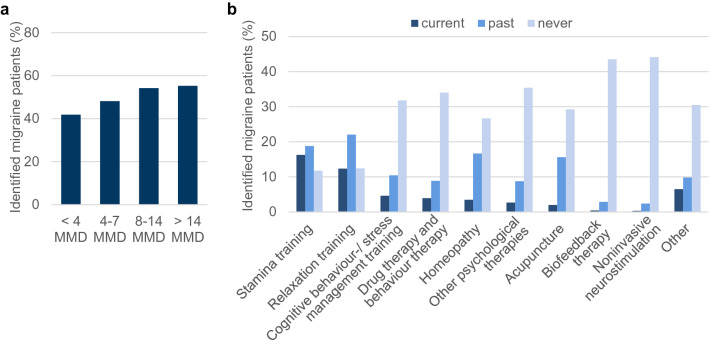
Drug-free prophylactic treatments. (**a**) Graph depicts the percentage of migraine patients that have already tried drug-free prophylactic treatment depending on their MMD. (**b**) Chart shows the percentage of patients that have never tried (light blue), have tried in the past (blue) or are currently using (dark blue) different kinds of drug-free prophylactic treatments. Stamina training includes for example jogging, swimming, or Nordic walking. Behaviour therapy comprises relaxation training, cognitive behaviour therapy, biofeedback therapy or stress management training. “Other” means other non-drug therapy methods for migraine prophylaxis.

47% (N = 10,068) of all identified migraine patients had previously tried drug-free prophylactic treatment with an increasing incidence depending on the number of MMD (Fig. 5A). At the time of the survey, most patients were carrying out stamina training (N = 3499; 16% of all identified migraine patients), followed by relaxation training (N = 2652; 12% of all identified migraine patients) (Fig. 5B). In general, more women than men have tested drug-free prophylactic treatments, and the number of patients that have tried drug-free prophylactic treatments was higher among patients who have already discontinued at least one prophylactic drug therapy (Supplementary Figure S3).

## Discussion

EPISCOPE is the largest survey among migraine patients in Germany to date and provides insights into clinical aspects and reasons for insufficient use of medical care by migraine patients in Germany.

EPISCOPE was performed as an online survey, advertised specifically among people who were identified as potential migraine patients via a Facebook algorithm. The advertisements were designed to appeal to people’s interest towards migraine or their willingness to help improve awareness of migraine disease, and thus resulted in an interest-guided study population. As seen before in other online studies^6^, the population questioned was younger than migraine populations seen in clinical trials^7–9^ or population based studies^2^. However, Lipton et al. previously showed that the age difference between participants of online and mail surveys did not have a differential effect on the results concerning disease burden and medical need^6^. EPISCOPE invited all migraine patients to participate independent of their MMDs and previous use of migraine-specific prophylaxis. EPISCOPE therefore expands the findings of other studies, which were restricted to patients with multiple treatment failures, high medical need, or had more than four MMDs^10^.

This study analysed different aspects of a patient’s journey. The first step of this journey is awareness of the disease. The study revealed that even in a population presumably biased by interest towards migraine there is still a substantial percentage of people who may not be aware of their disease. These results are in line with previous studies in Germany^11^ and France^12^. Part of this problem is probably related to patients disbelieving or even stigma: a social construct that serves to promote inequity for patients afflicted with various disease states^13^. Stigma carries negative social consequences but also impacts medical care of those who are stigmatised. Stigmatised patients are less likely to seek care. Indeed, the EUROLIGHT study clearly demonstrated that only a small part of people with migraine in Europe received adequate medical care ^2^. An earlier study in Turkish immigrants in Germany has shown that social disparities influence access to medical care, too^14^. Thus, poor awareness of people with migraine is one of the factors why they do not seek medical care.

Deficiencies in the available system of medical care might be another obstacle. Migraine care should be initiated and maintained in primary care for most affected individuals. In Europe, primary care can meet the needs of the majority of individuals who consult their primary care physician because of headaches^15^ since most of these individuals suffer from either migraine or tension-type headache. General practitioners require basic skills to diagnose migraine, exclude secondary causes, and initiate treatment^16,17^. Patients mostly with treatment-resistant or refractory migraine, rare headache disorders or medication overuse headache may require referral to specialist care. Benefits from specialist care are likely attributed to higher levels of expertise and availability of a multidisciplinary team. However, structured specialist services are scarce even in developed countries, e.g. Germany^15^. In addition, existing specialist services are often limited by long delays from the point of referral to consultation which is only to the detriment of the affected patients. In our study, the long waiting time was one of the most frequent reasons for not seeing a doctor, especially a specialist. Besides that, young patients in particular were uncertain about which health care professional to consult, which hints at a lack of awareness and further need for education of the German migraine population. Nevertheless, the proportion of patients who have received a migraine diagnosis by a doctor and the proportion of patients who consulted a doctor in our study were higher than the average reported in the European Community^2^, which was probably due to the bias in our patient cohort discussed above. It is also not surprising that migraine burden, e.g. high frequency of migraine attacks, was the major driving force for seeking medical consultation.

Similarly, severely affected patients were better informed and more willing to take acute and preventative medication. Our study demonstrated once again that even in a European country like Germany, with a well-developed system of medical care, the quality of treatment received by surveyed patients with headache was poor. More than 90% of patients used medications to treat migraine attacks but only a small proportion used preventative medication continuously. The German guidelines recommend starting preventative medication in patients with more than 4 MMD, especially in those with more than 8 MMD^18^. In our study, even in the group of patients with greater than or equal to 15 MMD half of them have never tried preventive treatment. Preventive medication was more frequently given by specialists than general practitioners. This finding is not specific for Germany but in line with previous reports^2^ that show broad differences in the education of physicians, as demonstrated by large discrepancies between general practitioners and neurologists concerning their prescription rate of preventive migraine treatment. In addition, among patients who have decided to try preventative drug therapy the adherence to this treatment was very low. These results concur with what has been described by Hepp et al.^19^ and illustrate again that many patients are not satisfied with the existing standard medications. The main reasons for discontinuation were dissatisfaction with the efficacy of the preventative treatment or side effects, which reinforces the need for new efficient prophylactic treatments with fewer side effects and an improved efficacy. It has to be kept in mind that EPISCOPE was conducted only a few months after the first representative of a new class of migraine prophylaxis, the calcitonin gene-related peptide (CGRP) pathway targeting monoclonal antibody erenumab, was available in Germany and we therefore assume that the vast majority had not tried this new drug at the time of the survey. It will therefore be very interesting to see if this new class of prophylaxis will be able to better satisfy the patients’ needs with regard to safety and efficacy which is already being investigated in a recently closed trial (NCT03828539).

Besides the preventive care there is also need to further investigate the use of acute medication (RX, OTC), potential barriers of their use, the perceived effectiveness and their potential as another approach to improve migraine care.

In the EPISCOPE survey the consulted migraine patients receive insufficient medical care; however, there are some limitations of the study that should be considered. First, it has to be kept in mind that the recruitment via targeted online advertisement resulted in an interest-guided study population and thus the population questioned was younger compared to other online studies^6^. Therefore, older migraine cases may be underrepresented in this study. Furthermore, this study does not provide any information on the medical care of people unaware of their own suffering from migraine or reasons for the lack of their awareness. Second, this study used the three item ID migraine screener, which is a well-established and reliable screening tool for migraine^20^. However, it must be remembered that this does not equal a validated migraine diagnosis as per ICHD-3 criteria. Around 25% of the participants who stated that they were suffering from migraine, did not have a medical diagnosis or were not sure whether a migraine had been diagnosed previously. Although these participants had a positive ID Migraine screener result, this population could include a proportion of patients that does not suffer from migraine but another headache disorder. Third, EPISCOPE did not capture the disease burden of migraine patients beyond disease duration and number of MMD. Further studies will be necessary to assess the correlation of medical care and quality of life parameters and to investigate how new migraine prophylaxes will affect disease burden. Fourth, the analysis eliminated individuals from the sample who met ID-Migraine symptom criteria for migraine but who did not consider themselves to have migraine. Therefore, identification of reasons for underdiagnosis and undertreatment are limited to those who believe they have migraine, and which is confirmed by the ID Migraine screener. Furthermore, as the migraine days are not confirmed by a symptom diary, the number of migraine days may be overestimated, and the number of total headache days may be underestimated. Fifth, there may be a response and recall bias in a highly stigmatized condition as migraine.

EPISCOPE is the largest survey that has been conducted among the whole migraine patient population in Germany so far. Although Germany is a rich country with a well-developed medical care system, the survey demonstrates insufficient medical care for the consulted migraine patients. Here, we identified three major reasons for this situation that need to be addressed in order to improve the medical care situation of these migraine patients. First, lack of patient education, which affects awareness of the disease and consultation of medical health care professionals. Second, limited access to specialist care including long waiting times which deters migraine patients from consultation and consequently prevents successful treatment of more severely affected migraine patients. Lastly, the limited tolerability and efficacy of oral non-specific preventive therapies, which are currently the standard of care therapy in Germany, may hinder migraine patients from trying a prophylactic therapy and those who do try may have a low treatment adherence due to the aforementioned limitations. Thus, we need earlier access to targeted preventive therapies with a better tolerability such as antibodies targeting the CGRP pathway.

## Methods

The EPISCOPE survey was performed from January to March 2019. We approached people in Germany suffering from migraine through a targeted media outreach campaign. The following advertising channels were used for the outreach campaign: social media advertising (Facebook/Instagram), newsletter and search engine advertising.

As EPISCOPE was an anonymous online survey and anonymous surveys do not require ethical approval according to German law. This survey was performed in accordance with the Helsinki Declaration of 1964 and its later amendments.

The survey was rolled out by Clariness GmbH and was compliant with ethics guidelines. Patients were informed about data storage, processing, and publication, and their informed consent was obtained. Before survey start, respondents had to confirm the informed consent on the landing page of the survey. The informed consent explained the intent of the survey. It also described how much time the survey will probably take and that the survey is anonymous, and the answers cannot be linked to the participants. The survey software is hosted on the fully secured server environment of Clariness GmbH in Germany which guarantees full compliance with all legal requirements for the protection of the survey answers. The survey questions and response options were developed together by Clariness GmbH and the authors. First, a text document was created in Word, in which all questions and answer options were listed. For each question there was also the information in which question format the question should be asked (single choice, multiple choice, etc.). The document also described the conditions under which a question should be asked. This document was proofread and confirmed by the authors before being entered into the survey software. A Patient Insights Manager set up the developed questionnaire. Before the survey was released, a quality check was performed. A second Patients Insights Manager checked whether the settings were correct, and the questions were set up according to the document. Using a test link the authors performed a final test, followed by survey activation. Survey pre-testing with a panel of migraine patients was not done. Survey respondents didn’t receive any incentive for their participation.

To avoid multiple entries of the same respondent, a cookie was placed in the internet browser of the participant after completion. When the respondent tried to start the survey again with the same internet browser, an error message was shown. Since fully anonymous, open surveys can’t completely prevent multiple entries. Only fully completed questionnaires were used as data basis for the survey reporting. Almost all questions displayed to the participants were made mandatory. So, it was technically not possible to continue the survey without providing an answer. Only exceptions on purpose: Q06 and Q07 (questions about region and zip code).

We have targeted users on Facebook/Instagram who are interested in migraine topics (e.g. “Prevention of migraines”, “Migraine News”). Participants’ attention was attracted by different ad formats with a heading that was always connected to the catchphrase “Migraine”. Advertisement wording varied in order to reach a broad patient population: “Your opinion is important for us”, “Your experiences are important”, “You are invited to participate in a migraine survey”, “You can help us to understand patients with migraine better” or “New migraine survey is online”. To better control the demographic aspect, we have segmented the interest targeting by gender in two ad groups (female/male). After people who were interested clicked on the advertisement, they were redirected to the landing page, where they were informed about the purpose of the survey and aspects of data protection. People who wanted to participate in the survey could do so by clicking on the call-to-action button (“Start Survey”). To support the Facebook algorithm's purpose of showing our ads to users who are more likely to complete the survey, the Facebook conversion pixel was implemented on our website.

The newsletter was addressed to persons subscribed to the ClinLife “migraine” newsletter (Opt-in).

The survey comprised five domains and 36 questions, which were designed to obtain information about every step of the patient journey (Supplementary Table S2). The first domain covered general baseline characteristics of all participants such as sex, age, and occupation. The second domain collected information on clinical details, including onset and prevalence of their migraine and the ID Migraine screener (for ID Migraine questions see Supplementary Table S1) in order to identify those participants who did indeed suffer from migraine. The ID Migraine screener is a brief, self-administered questionnaire for patients with headache complaints asking for disability, nausea and sensitivity to light (photophobia) (German version, sensitivity of 99% and a specificity of 68%)^20,21^. In the next domain, participants were asked about the medical care they had, starting from questions about their diagnosis to their current treatment situation. The fourth domain covered the topic of acute medication, including frequency of migraine-specific medication usage and patient education with regard to medication overuse. In the last domain, the usage and patient view on prophylactic treatment was assessed, covering both drug and drug-free prophylactic treatments.

Participants who were older than 18 years, lived in Germany and suffered from migraine confirmed by diagnostic questions of the survey (inclusion criteria) were entered for final analysis.

### Analysis and statistics

Participants who stated that they did not suffer from migraine or were unsure if they did were excluded from answering the remaining questions—even if some of them were indeed identified as migraine patients by the ID Migraine screener—as they did not consider themselves as migraine patients and therefore most likely never followed up on their disease.

Participants were grouped based on the headache frequency in the three months before the survey (monthly migraine days, MMD) in the four groups: low-frequency (< 4 MMD), medium-frequency (4–7 MMD), and high-frequency episodic migraine (8–14 MMD), and chronic migraine (≥ 15 MMD), based on the age (18–25, 26–35, 36–45, 46–55, 56 + years) or based on the number of prophylactic treatments that they have tried and discontinued (none tried, still on first prophylaxis, 2/3/4/ ≥ 5 discontinued). The analysis of the monthly migraine days of the survey participants is based on question Q08 (“How often have you on average suffered from migraine within the last 3 months?”).

Categorical variables were summarised as proportions (%) and frequencies (N), and continuous variables as means ± standard deviation (SD). The data was analysed using Tableau Reader. The analysis is purely descriptive, no statistical comparisons were performed.

Figures were generated using Microsoft PowerPoint.

### Consent to participate

Patients were informed about data storage, processing, and publication and their informed consent was obtained. Consent to publish is not applicable for this survey.

## Supplementary Information

Supplementary Information.

## Data Availability

The datasets used and/or analysed during the current study are available from the corresponding author upon reasonable request.
