# Supplementary material for: Results of the gErman migraine PatIent Survey on medical Care and prOPhylactic treatment Experience (EPISCOPE)
Source: Sci Rep. 2022 Mar 17;12:4589. doi: 10.1038/s41598-022-08716-w (PMC8931117; doi:10.1038/s41598-022-08716-w)
Supplement: Supplementary file 1 — Supplementary Information. [file 41598_2022_8716_MOESM1_ESM.pdf]

Supplementary Figure S1

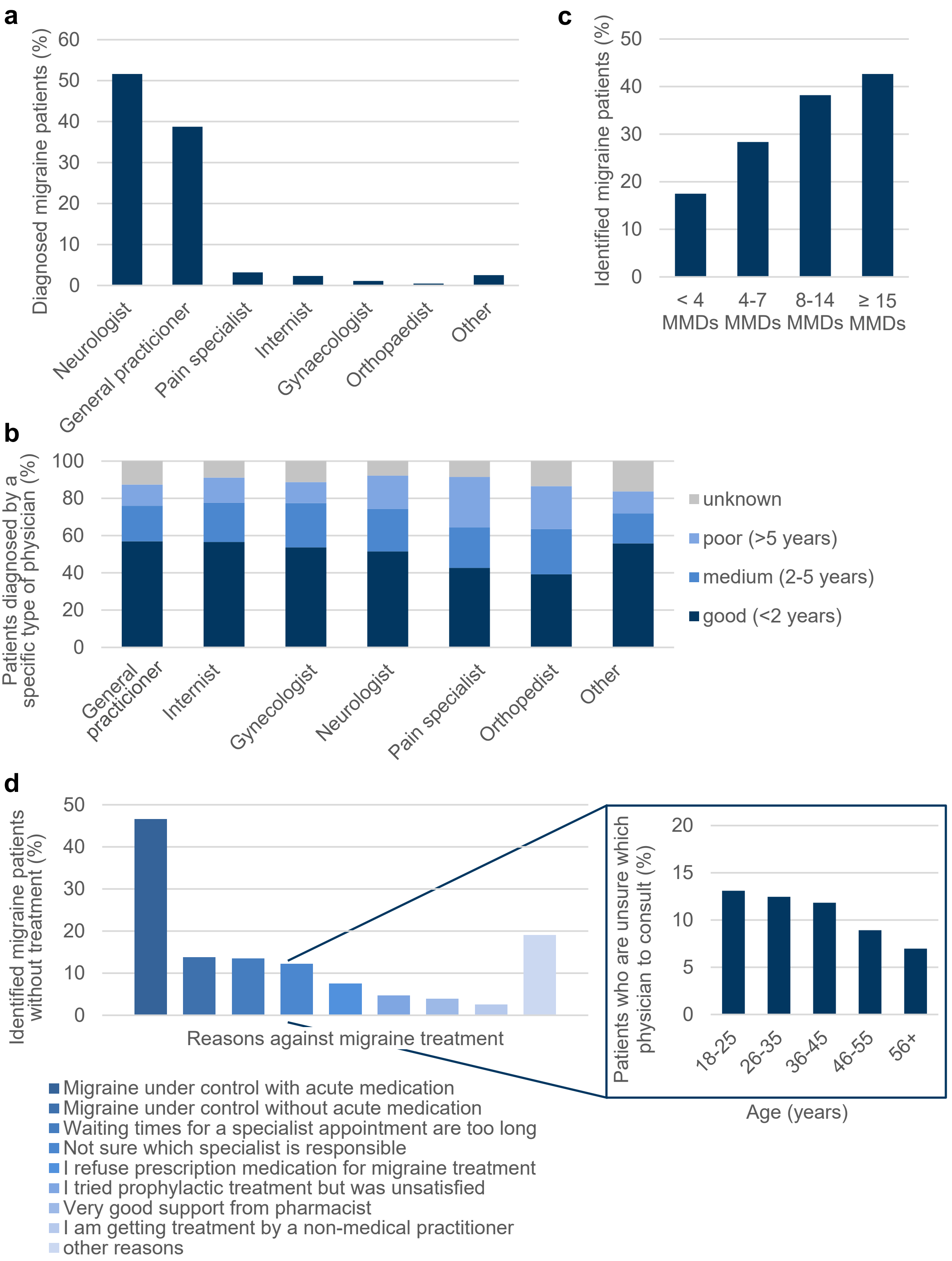

(a) Graph shows which physicians diagnosed patients with migraine (N = 15,994 diagnosed patients). **(b)** Shown are the proportions of patients that were diagnosed within a good (<2 years), medium (2-5 years) or poor (>5 years) time frame after diagnosis dependent on the type of physician that documented the diagnosis. Number of patients diagnosed by: general practitioners: N = 6,198; internist: N = 371; gynaecologist: N = 177; neurologist: N = 8,256; pain specialist: N = 513; orthopaedist: N = 74; other physician: N = 405. **(c)** Indicated is the percentage of all identified migraine patients who are currently receiving medical treatment for their migraine depending on the number of their MMD. Number of patents currently in therapy in the respective groups: <4 MMD: N = 1,736; 4-7 MMD: N = 1,785; 8-14 MMD: N = 1,266; ≥15 MMD: N = 842. **(d)** Graph shows ranked reasons for not seeking medical advice. Those asked were all patients who are currently not seeing a physician (N = 15,875). On the right, the percentage of patients who were unsure which physician to consult was indicated dependent on the patient's number of MMD. 18-25 years: N = 1,037 out of 7,964 patients; 26-35 years: N = 571 out of 4,757 patients; 36-45 years: N = 288 out of 1,977 patients; 56-55 years: N = 82 out of 933 patients; >55 years: N = 16 out of 244 patients.

Supplementary Figure S2

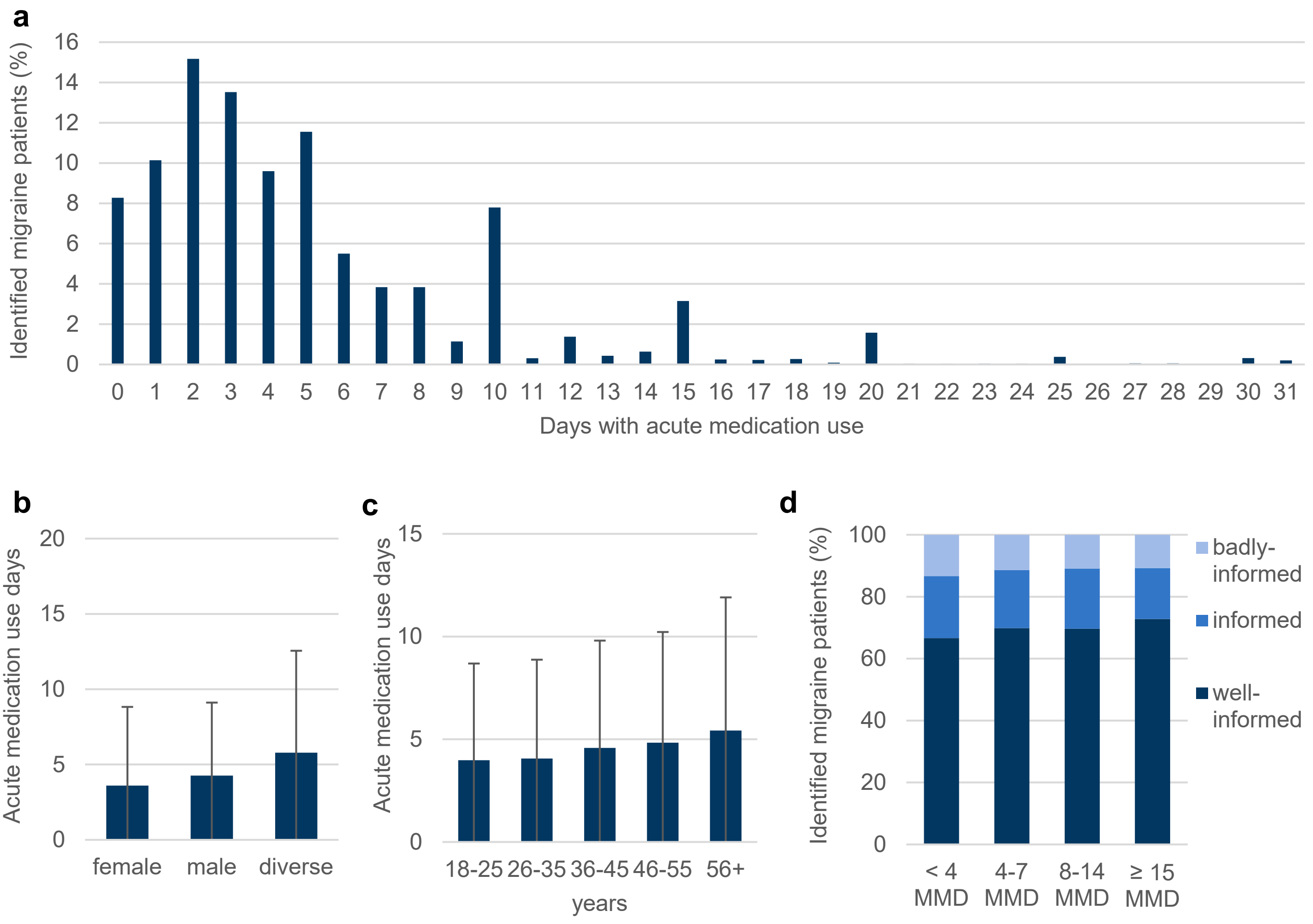

(a) Shown is the distribution of monthly days with acute medication use across all identified migraine patients (N = 21,504). **(b + c)** Graphs show the number of monthly days with acute medication use dependent on gender **(b)** and age **(c)**, respectively. Shown are means +/- SD. Total patient numbers: female: N = 18,390; male: N = 3,077; diverse: N = 37; 18-25 years: N = 10,384 patients; 26-35 years: N = 6,358 patients; 36-45 years: N = 2,810 patients; 56-55 years: N = 1,468 patients; >55 years: N = 466 patients. **(d)** Graph shows the percentage of identified migraine patients that feel well-informed (dark blue), informed (blue), and badly informed (light blue) about medication-overuse depending on the number MMD. Total patients per group: <4 MMD: N = 9,920; 4-7 MMD: N = 6,297; 8-14 MMD: N = 3,313; ≥15 MMD: N = 1,974.

Supplementary Figure S3

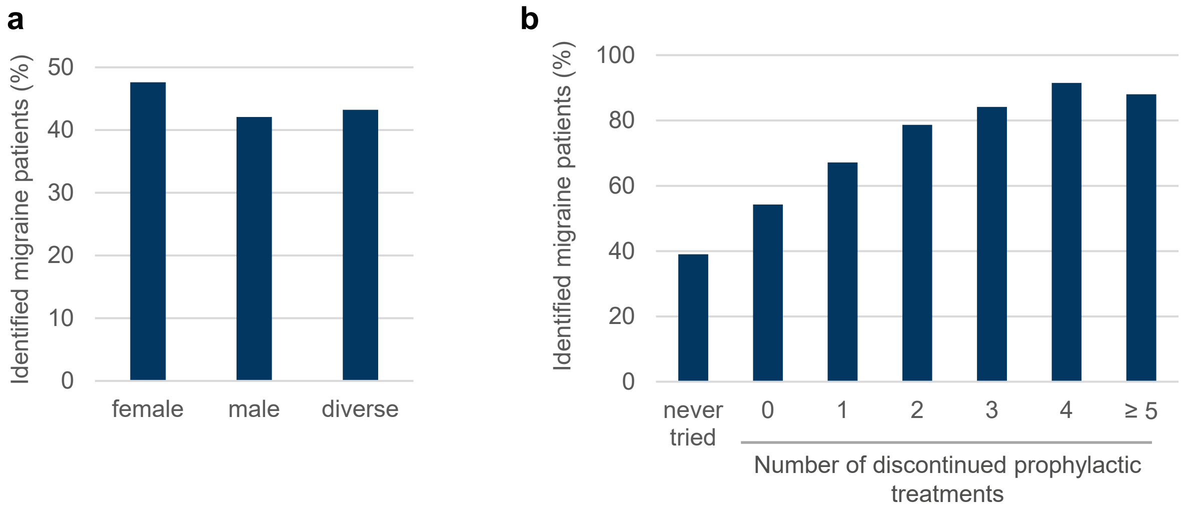

(a) Graphs show the percentages of identified migraine patients that have ever tried non-drug prophylactic treatment dependent on age. Total number of patients: female: 8,757 of 18,390; male: 1,295 of 3,077; diverse: 16 of 37. **(b)** Percentages of identified migraine patients that have ever tried non-drug prophylactic treatment dependent on the number of discontinued prophylactic treatments. Total patient numbers: never tried prophylactic treatment: N = 5,701 of 14,614; 0 discontinued: N = 1,877 of 3,460, 1 discontinued: N = 1,479 of 2,203; 2 discontinued: N = 4517 of 657; 3 discontinued: N = 239 of 284; 4 discontinued: N = 86 of 94; ≥5 discontinued: N = 169 of 192.

Supplementary Table S1: ID-Migraine questions to identify patients who suffer from migraine.

	**ID-Migraine questions**
1	“Have you ever suffered from nausea when having a headache?”
2	“Are you more sensitive to light when having a headache?”
3	“Has a headache ever restricted your ability to work, study or fulfil any other obligations for at least one day?”

Additional file 1: EPISCOPE Questionnaire

	Questions	Selection Choices
1	Please state your age.	- 5-115 years
2	Please select your sex.	- Male<br>- Female<br>- Diverse
3	Are you suffering from migraine?*	- Yes<br>- No<br>- Not sure

* The answer to this question determined, which of the following questions were asked:

- everyone was asked to answer questions 4–7
- “yes” → questions 8-17, 21 (ID Migraine), 22, 23–36 were asked in the here indicated order
- “no”/”not sure” → only questions 18–22 (including the ID Migraine questions) were asked in the here indicated order

4	Please select your occupation.	- School<br>- Student<br>- Employed<br>- Not employed due to migraine<br>- Not employed due to other reasons<br>- Early retirement due to migraine<br>- Retired
5	Where do you live?	- Germany<br>- Other country
6	Please select the state where you live.	- Berlin<br>- Bayern (Bavaria)<br>- Niedersachsen (Lower Saxony)<br>- Baden-Württemberg<br>- Rheinland-Pfalz (Rhineland-Palatinate)<br>- Sachsen (Saxony)<br>- Thüringen (Thuringia)<br>- Hessen<br>- Nordrhein-Westfalen (North Rhine-Westphalia)<br>- Sachsen-Anhalt (Saxony-Anhalt)<br>- Brandenburg<br>- Mecklenburg-Vorpommern (Mecklenburg-Western Pomerania)<br>- Hamburg<br>- Schleswig-Holstein<br>- Saarland<br>- Bremen
7	What is your postcode?	
8	How often have you on average suffered from migraine within the last 3 months?	

9	Since when have you suffered from migraine?	-	1910-2018
10	Has a physician diagnosed you with migraine?	-<br>-<br>-	Yes<br>No<br>Not sure
11	What kind of physician has diagnosed your migraine?	-<br>-<br>-<br>-<br>-<br>-	General practitioner<br>Neurologist<br>Pain specialist<br>Gynaecologist<br>Orthopaedist<br>Other physician
12	How long did it take from your first migraine symptoms to diagnosis?	-<br>-<br>-<br>-<br>-<br>-<br>-	< 1 year<br>1-2 years<br>2-3 years<br>3-4 years<br>4-5 years<br>>5 years<br>Don't know
13	Do you think that you are well-informed about medication that is available for migraine?	-<br>-<br>-	I am well-informed.<br>I am informed but my knowledge could be better.<br>No, I am not sufficiently informed.
14	Are you currently seeing a doctor for your migraine?	-<br>-	Yes<br>No
15	What kind of physician are you seeing to treat your migraine?	-<br>-<br>-<br>-<br>-<br>-	General practitioner<br>Neurologist<br>Pain specialist<br>Gynaecologist<br>Orthopaedist<br>Other physician
16	I am currently not being treated by a physician because:	-<br>-<br>-<br>-<br>-<br>-<br>-<br>-<br>-	I have the migraine under control with acute medication<br>I have the migraine under control without acute medication<br>Waiting times for a specialist appointment are too long<br>I am not sure which specialist is responsible<br>I refuse prescription medication for migraine treatment<br>Very good support from pharmacist<br>I tried prophylactic treatment but was unsatisfied<br>I am getting treatment by a non-medical practitioner<br>Other reasons
17	To which kind of physician would you go if you decided to get treatment for your migraine?	-<br>-<br>-	General practitioner<br>Neurologist<br>Pain specialist

		- Gynaecologist<br>- Orthopaedist<br>- Naturopathic practitioner<br>- Other physician
18	Have you ever suffered from migraine?	- Yes<br>- No
19	Have you suffered from headache within the last year?	- Yes<br>- No
20	How many headache days did you have last months?	- 0-30 days
21	ID Migraine (see Additional file 2): Please indicate if the following statements apply to you.	- Yes<br>- No
22	If you think about the last three months: how often did you have the "worst headache", i.e. the headache that bothers and affects you the most?	
23	Have you ever taken any medication for migraine prophylaxis?	- Yes<br>- No
24	Have you ever discontinued prophylactic migraine treatments?	- No<br>- One migraine prophylaxis<br>- Exactly two migraine prophylaxis<br>- Exactly three migraine prophylaxis<br>- Exactly four migraine prophylaxis<br>- 5 or more migraine prophylaxis discontinued
25	Why have you discontinued a migraine prophylaxis?	- Treatment did not help at all<br>- Side effects<br>- Treatment did not help sufficiently<br>- Incompatibilities with other drugs<br>- Other reasons
26	Are you currently taking prophylactic migraine medication?	- Yes<br>- No
27	Do you tolerate your current migraine prophylaxis?	- Yes, very well.<br>- Yes, with side effects.<br>- No, due to side effects.<br>- No, due to insufficient efficacy.<br>- No, due to daily intake.<br>- No, due to other reasons.
28	I do not take migraine prophylaxis because:	- I don't know enough about medication for preventive migraine treatment.<br>- I have the migraine under control with acute medication<br>- I refuse prescription medicine for preventative migraine treatment.<br>- I refuse preventative migraine treatment, as it has to be taken daily.<br>- I refuse these drugs due to side effects.<br>- None of the above.

29	On how many days did you take pain-relieving medication due to migraine or headaches?	- 0 days<br>- 0-3 days<br>- 4-10 days<br>- 11-15 days<br>- More than 15 days
30	Do you feel informed about medication overuse?	- I know when medication overuse can occur and its risks.<br>- I have heard about medication overuse but I don’t know much about it.<br>- I know (almost) nothing about medication overuse.
31	Where do you get your information about medication overuse?	- Physician<br>- Pharmacist<br>- Naturopathic practitioner<br>- Patient organization<br>- Internet<br>- Television<br>- Print media (e.g. newspaper)<br>- Social media (e.g. Facebook)<br>- Others
32	Have you ever tried anything to prevent migraine except medication?	- Yes<br>- No
33	What kind of non-medication migraine prophylaxis have you tried or are you currently using?	- Stamina training<br>- Relaxation training<br>- Cognitive behaviour/ stress management therapy<br>- Drug therapy & behaviour therapy<br>- Homeopathy<br>- Other psychological therapies<br>- Acupuncture<br>- Biofeedback therapy<br>- Non-invasive neurostimulation<br>- Others
34	How often have you used the respective non-drug prophylactic treatment or are you planning to use this year?	- 1<br>- 2-3<br>- 4-5<br>- 6-10<br>- 11-15<br>- 16-20<br>- More than 20
35	What would be important for you concerning a new prophylactic migraine drug?	- Good tolerability (few side effects)<br>- Fast onset of effect<br>- Good efficacy<br>- Low application rate (monthly instead of daily)<br>- No interactions with other drugs or contraindications (e.g. pregnancy)<br>- Simple application

		- Additional Information material including instructions for proper use<br>- Additional Information material including information about the disease<br>- Supportive service programme, e.g. training for proper use
36	Where do you get information about migraine and treatment options?	- Physician<br>- Nurse<br>- Pharmacy<br>- Internet (specific websites about migraine)<br>- Internet (discussion forum)<br>- Social media (e.g. Facebook)<br>- Online communities<br>- Migraine apps<br>- Other migraine patients<br>- Migraine support groups, e.g. “Migräneliga”/”Deutsche Schmerzliga”<br>- None of the above<br>- I do not inform myself about migraine.
